# The Microscopical Appearance of the Heart and Kidney in the Case Reported by Prof. N. S. Davis, M.D.

**Published:** 1874-04-15

**Authors:** Lester Curtis


### THE MICROSCOPICAL APPEARANCE OF THE HEART AND KIDNEY IN THE CASE REPORTED BY PROF. N. S. DAVIS, IN THE PRESENT NUMBER OF "THE EXAMINER."

THE piece of heart which I received from Dr. Davis presented, on the outside, simple atheromatous and calcareous degeneration. The muscular fibres appeared healthy. The kidney presented a mottled appearance, part being of a cream-color, other portions being of a natural color, except much paler. I took two small pieces of this kidney and placed them in a weak solution of chromic acid, to harden. After a day or two, I cut some thin sections, both in a longitudinal and a transverse direction, and stained them in an alkaline solution of carmine. On examining the sections with the microscope, the whole field appeared confused, and it was only after repeated and prolonged examination that I was enabled to make out anything at all satisfactory. This was particularly the case over the grayer portions. The cause of this indistinctness was the infiltration of the organ with a granular substance. In some places this granular substance was replaced by round bodies resembling, in size and appearance, pus corpuscles; in other places there were collections of round bodies from one-third to one-half the diameter of the former; neither of these collections had well-defined boundaries. The edges of some of the sections, which were extremely thin, showed, where the granular material had been washed out, that the connective tissue of the kidney was somewhat thickened, and contained many more muscular points than in health. The Malpighian tufts were, in many places, contracted down into little compact knots, of cicatricial-like tissue. The uriniferous tubules were filled with a granular material; the cells lining them had lost their distinctive characteristics, and were cloudy and opaque. Most of the straight tubules were wasted to mere irregular, nodulated cords.

These appearances do not correspond altogether with any specimen that I have met before, or with any description that I have seen published. I should dislike, at present, to give a decided opinion as to their nature; they correspond, however, more closely with what Rindfleisch calls *cellular hypertrophy of the connective tissue*, than anything else with which I am acquainted.

LESTER CURTIS, M.D.

